# Taurine Protects against Postischemic Brain Injury via the Antioxidant Activity of Taurine Chloramine

**DOI:** 10.3390/antiox10030372

**Published:** 2021-03-02

**Authors:** Song-I Seol, Hyun Jae Kim, Eun Bi Choi, In Soon Kang, Hye-Kyung Lee, Ja-Kyeong Lee, Chaekyun Kim

**Affiliations:** 1 Department of Anatomy, Inha University School of Medicine, Incheon 22212, Korea; ssie8878@naver.com (S.-I.S.); marrina@hanmail.net (H.-K.L.); 2 BK21, Program in Biomedical Science & Engineering, Inha University, Incheon 22212, Korea; zzang4128z@naver.com (H.J.K.); chldmsql777@naver.com (E.B.C.); 3 Laboratory of Leukocyte Signaling Research, Department of Pharmacology, Inha University School of Medicine, Incheon 22212, Korea; round001@hanmail.net; 4 Convergent Research Center for Metabolism and Immunoregulation, Inha University, Incheon 22212, Korea

**Keywords:** taurine, taurine chloramine (Tau-Cl), middle cerebral artery occlusion (MCAO), neutrophils, myeloperoxidase (MPO), antioxidant enzymes

## Abstract

Taurine is ubiquitously distributed in mammalian tissues and is highly concentrated in the heart, brain, and leukocytes. Taurine exerts neuroprotective effects in various central nervous system diseases and can suppress infarct formation in stroke. Taurine reacts with myeloperoxidase (MPO)-derived hypochlorous acid (HOCl) to produce taurine chloramine (Tau-Cl). We investigated the neuroprotective effects of taurine using a rat middle cerebral artery occlusion (MCAO) model and BV2 microglial cells. Although intranasal administration of taurine (0.5 mg/kg) had no protective effects, the same dose of Tau-Cl significantly reduced infarct volume and ameliorated neurological deficits and promoted motor function, indicating a robust neuroprotective effect of Tau-Cl. There was neutrophil infiltration in the post-MCAO brains, and the MPO produced by infiltrating neutrophils might be involved in the taurine to Tau-Cl conversion. Tau-Cl significantly increased the levels of antioxidant enzymes glutamate–cysteine ligase, heme oxygenase-1, NADPH:quinone oxidoreductase 1, and peroxiredoxin-1 in BV2 cells, whereas taurine slightly increased some of them. Antioxidant enzyme levels were increased in the post-MCAO brains, and Tau-Cl further increased the level of MCAO-induced antioxidant enzymes. These results suggest that the neutrophils infiltrate the area of ischemic injury area, where taurine is converted to Tau-Cl, thus protecting from brain injury by scavenging toxic HOCl and increasing antioxidant enzyme expression.

## 1. Introduction

Taurine (2-aminoethansulfolic acid), the decarboxylation product of cysteine, is one of the most abundant free amino acids in the animal kingdom. It has various physiological functions including osmoregulation, membrane stabilization, calcium mobilization, neurotransmission, reproduction, inflammation, and detoxification [1,2,3]. Taurine is ubiquitously distributed in mammalian tissues, with the highest levels found in the heart, brain, and leukocytes [1]. Taurine levels differ in different regions of the brain [4]. In the adult brains of mice, rats, and humans, 8.6, 4.4, and 1.4 µmol/g wet weight of taurine, respectively, were detected, and these were 2–4-fold higher in the newborn brain than in the adult brain [5], which indicates that taurine concentration is the highest in developing brain and decreases with development. Taurine has neuroprotective activity in various brain injury models, including ischemic stroke and inflammation [6,7,8,9,10]. Taurine reduced apoptotic protein expression in ischemic injury [11] and maintained intracellular Ca^2+^ homeostasis [12], thereby attenuating apoptotic neuronal death [7]. Taurine also induced protection against endoplasmic reticulum (ER) stress by reducing the expression of the components of the ER stress pathway and by suppressing excessive reactive oxygen species (ROS) production [13]. Moreover, taurine suppressed proinflammatory cytokine production and neutrophil infiltration [8] and prevented hemorrhage by reducing intravascular deposition of fibrin/fibrinogen and platelets [14].

Neutrophils, the first-line immune defense cells, are the most abundant leukocytes in the blood. Neutrophils infiltrate in the regions of inflammation or infected tissues and engulf invading microorganisms, killing them with oxidants and microbicidal proteins. Neutrophils contain large amounts of taurine, constituting 30–75% of the total free amino acids. Human neutrophils contain 10–70 mM taurine, whereas plasma contains an average of 50 µM taurine [15,16]. In activated neutrophils, taurine reacts with hypochlorous acid (HOCl), which is released by the myeloperoxidase (MPO) system, to generate taurine chloramine (Tau-Cl) [17]. Thus, taurine ameliorates inflammation by eliminating highly toxic HOCl. Moreover, Tau-Cl suppresses the production of many proinflammatory mediators and upregulates antioxidant enzymes, thus protecting cells at the site of inflammation from inflammatory cytotoxicity [2,3]. Tau-Cl increases the cytosolic levels and nuclear translocation of nuclear factor E2-related factor (Nrf2) and the binding of Nrf2 to the antioxidant response element [18,19]. Nrf2 regulates the expression of antioxidant enzymes, such as heme oxygenase (HO), NADPH:quinone oxidoreductase (NQO), glutathione peroxidase (GPx), peroxiredoxin (Prx), and glutamate–cysteine ligase (GCL) [20,21]. GCL catalyzes glutamate and cysteine ligation to synthesize glutathione, which regulates the redox balance and prevents damage caused by ROS. HO catalyzes the oxidative degradation of free heme and releases ferrous iron, CO, and biliverdin/bilirubin. Therefore, the Tau-Cl-induced elevation of HO-1 produces bilirubin and CO, which eliminates toxic hydroxyl radicals and prevents ROS production, respectively [3]. NQO is an obligate two-electron reductase that catalyzes the reduction of quinones to hydroquinones using flavin adenine dinucleotide as a cofactor [22], and it is commonly proposed to be involved in the mechanism of antioxidation and detoxification. Prx reduces peroxide, and its activity is associated with the removal of hydrogen peroxide, organic hydroperoxides, and peroxynitrite. Prx is critical for protecting cellular components from oxidative damage [23,24].

Stroke is the third leading cause of mortality and the leading cause of disability worldwide. Ischemic brain injury is induced by a complex series of pathological events during stroke, which are initiated by excitotoxicity- and Zn^2+^-toxicity-dependent massive neuronal death. This acute neuronal damage is followed by a second round of neuronal injury in the neighboring regions [25]. Postischemic inflammation, apoptosis, and oxidative stress, which occur from few hours to days after the primary ischemic event, are associated with this delayed injury. In particular, oxidative stress contributes to the late stages of ischemic injury and to worse neurological outcomes [25,26]. ROS are produced during cerebral ischemia, perturbing the prooxidant–antioxidant balance and damaging cellular macromolecules, such as lipids, proteins, and nucleic acids [27]. In addition, oxidative stress indirectly causes cellular damage by inducing apoptosis and inflammation [27]. The neuroprotective effects of taurine have been reported in transient stroke animal models, such as the middle cerebral artery occlusion (MCAO) model [6,7,8,28,29,30,31].

The brain contains large amounts of taurine, and neutrophils infiltrate the ischemic brain. The abundant taurine reacts with HOCl produced by the MPO system in activated neutrophils to produce Tau-Cl. In the present study, we hypothesized that taurine exerts a neuroprotective effect to some extent through the action of Tau-Cl. We investigated this hypothesis using a rat MCAO model and BV2 microglial cells. The neutrophils infiltrated in the MCAO brain. Tau-Cl reduced MCAO-induced infarct volume and neurological deficits. Tau-Cl increased the expressions of antioxidant enzymes. Accordingly, the results suggest that taurine is converted to Tau-Cl by the MPO released from neutrophils in the postischemic brain, and Tau-Cl increases the antioxidant enzymes. Therefore, taurine protects brain injury by scavenging toxic HOCl and increasing antioxidant enzymes in the MCAO brain.

## 2. Materials and Methods

### 2.1. Reagents and Tau-Cl Synthesis

Antibodies against HO-1 (Enzo, East Farmingdale, NY, USA), glutamate–cysteine ligase modifier (GCLM) subunit and MPO (Abcam, Cambridge, UK), NQO1 and Nrf2 (Santa Cruz Biotech, Santa Cruz, CA, USA), Prx-1 (Ab Frontiers, Seoul, Korea), and β-actin were purchased from commercial sources. Dulbecco’s modified Eagle’s medium (DMEM), fetal bovine serum (FBS), phosphate-buffered saline (PBS), penicillin, and streptomycin were purchased from HyClone (Logan, UT, USA). Oligonucleotides were purchased from Bioneer (Daejeon, Korea). Other chemicals were purchased from Sigma-Aldrich (St. Louis, MO, USA) unless indicated otherwise.

Tau-Cl was synthesized freshly on the day of use by adding equimolar amounts of NaOCl to taurine. The formation of Tau-Cl and its concentration were monitored by measuring UV absorption at 200–400 nm [32].

### 2.2. MCAO Procedure

Male Sprague–Dawley rats were housed under diurnal lighting conditions and allowed access to food and tap water ad libitum. All animal studies were conducted in strict accordance with the Guide for the Care and Use of Laboratory Animals published by the National Institute of Health (NIH, Bethesda, MD, USA, 2013) and the ARRIVE guidelines (http://www.nc3rs.org/ARRIVE (accessed on 31 January 2021)). The animal protocol used in this study was reviewed and approved by the University-Institutional Animal Care and Use Committee (INHA-IACUC) (approval Number INHA-180105-531-2). MCAO was carried out as previously described [33]. In brief, rats (250–300 g body weight) were anesthetized with 5% isoflurane in 30% oxygen/70% nitrous oxide and maintained using 0.5% isoflurane in the same gas mixture during surgery. Occlusion of the right middle carotid artery (MCA) was induced for 1 h by advancing a nylon suture (4-0; AILEE, Busan, Korea) with a heat-induced bulb at its tip (~0.3-mm diameter) along the internal carotid artery for 20–22 mm from the bifurcation of the external carotid artery. MCAO was followed by reperfusion for up to 2 days. The left femoral artery was cannulated for blood sampling to analyze PaO_2_, PaCO_2_, pH, and blood glucose concentrations (I-STAT; Sensor Devices, Waukesha, WI, USA). Regional cerebral blood flow (rCBF) was monitored using a laser Doppler flowmeter (Periflux System 5000; Perimed, Jarfalla, Sweden). Occlusion was considered successful if cortical CBF was reduced greater than 70% immediately after inserting an occluding suture. Animals were excluded if CBF was not reduced to less than 30% of baseline during MCAO or the blood flow was not restored during reperfusion. Both thermoregulated heating pad and heating lamp were used to maintain a rectal temperature of 37.0 ± 0.5 °C during surgery. Animals in the sham control group were operated on in a similar manner, but the MCA was not occluded. Animals were randomly allocated to one of four groups: MCAO, PBS-treated MCAO controls; MCAO + Taurine, taurine administered to MCAO; MCAO + Tau-Cl, Tau-Cl administered to MCAO; Sham, sham-surgery control group that underwent surgery but was not subjected to MCAO.

### 2.3. Intranasal Delivery

Intranasal administration was performed as previously described by Kim et al. [34]. In brief, rats were anesthetized with an intramuscular injection of a mixture of ketamine (3.75 mg/100 g body weight) and xylazine hydrochloride (0.5 mg/100 g body weight). Animals were then randomly divided into eight groups, as mentioned previously. A nose drop containing 0.1, 0.5, 1, or 2 mg/kg of Tau-Cl, 0.5 or 1 mg/kg of taurine, or PBS (10 μL) was carefully placed in each nostril of the anesthetized animals (supine 90° angle) using a pre-autoclaved pipet tip (T-200-Y, Axygen, CA, USA). The procedure was repeated until the entire dosage (total 10 μL) had been administered, at 2 min intervals between applications.

### 2.4. Infarct Volume Assessment

Rats were decapitated at 2 days post-MCAO, and whole brains were dissected coronally into 2 mm slices using a metallic brain matrix (RBM-40,000, ASI, Springville, UT, USA). Slices were immediately stained by immersion in 2% 2,3,5-triphenyl tetrazolium chloride (TTC) at 37 °C for 15 min and then fixed in 4% paraformaldehyde. Areas of infarcted tissue were measured using Scion Image (Frederick, MD, USA). To account for edema and shrinkage, areas of ischemic lesions were calculated as (contralateral hemisphere volume—(ipsilateral hemisphere volume—measured injury volume)). Infarct volumes were calculated (in mm^3^) by multiplying summed section infarct areas by section thickness.

### 2.5. Modified Neurological Severity Scores

Neurological deficits were evaluated using modified neurological severity scores (mNSSs) 2 days post-MCAO. The mNSS system comprises motor, sensory, balance, and reflex tests, and they are graded on a scale of 0–18, where higher scores represent more severe injury. Motor scores were determined by two tests: (1) suspending a rat by its tail, a score of zero or one was allocated for each sub-item (total score 0–3), that is, flexion of forelimbs, flexion of hind limbs, head movement > 10° with respect to the vertical axis within 30 s; (2) placing a rat on the floor, and scores from zero to three were allocated: 0—normal walking, 1—an inability to walk straight, 2—circling toward the paretic side, and 3—falling on the paretic side. Sensory tests included a placement test (score 0 or 1) and a proprioceptive test (score 0 or 1). The beam balance test was used to test balance, and scores from 0 to 6 were allocated as follows: 0, balance with a steady posture; 1, grasping the side of the beam; 2, hugging the beam with one limb off the beam; 3, hugging the beam and two limbs off the beam or spinning around the beam for 60 s; 4, attempting to balance on the beam but falling off within 20–40 s; 5, attempting to balance on the beam but falling off within 20 s; and 6, making no attempt to balance or hang on to the beam. Reflex test scores were determined by awarding scores to the following four items (total score 0–4): pinna reflex, 0 or 1; corneal reflex, 0 or 1; startle reflex, 0 or 1; seizures, myoclonus, or myodystony, 0 or 1.

### 2.6. Rotarod Test

Twenty-four hours before MCAO, rats were conditioned on a rotarod unit at a constant speed (3 rpm) until they could remain on the rotating spindle for 180 s. Each rat was subjected to rotarod testing at 5, 10, or 15 rpm at 2 days post-MCAO. Residence times on the rotarod were measured.

### 2.7. Hematoxylin and Eosin (H&E) Staining

Animals were anesthetized and subjected to cardiac perfusion with saline, followed by a 4% paraformaldehyde flush. Brains were soaked in the same fixative for 48 h at 4 °C, cut into appropriate portions (from bregma −1 to 3 mm coronally), and placed in embedding cassettes. The tissues were dehydrated by submerging in 70, 80, 90, and 100% alcohol for 1 h each, followed by 100% alcohol overnight at 20–22 °C. Tissue clearance was performed by immersion in xylene twice for 30 min each, followed by immersion three times in paraffin for 1 h each. Tissues were embedded into paraffin blocks, and 8 μm sections were obtained with a microtome. Sectioned tissue was deparaffinized in xylene twice for 3 min each, and rehydrated by submerging in 100, 90, 80, and 70% alcohol for 3 min. For H&E staining, slides were soaked in Mayer’s hematoxylin solution for 1 min, transferred into 0.1% acid alcohol to reduce background, and processed in eosin solution for 30 s.

### 2.8. Immunofluorescence Staining

Animals were euthanized at the indicated times after MCAO, and brains were fixed in 4% paraformaldehyde by transcardiac perfusion and then postfixed in the same solution overnight at 4 °C. Brain sections (20 μm) were produced using a vibratome, and immunological staining was performed as previously described [35]. The anti-MPO primary antibody was applied at 1:200 dilution. After washing with PBS containing 0.1% Triton X-100, sections were incubated with anti-rabbit IgG (Vector Laboratories, Burlingame, CA) in PBS for 1 h at 20–22 °C and visualized using the HRP/3,3′-diaminobenzidine system (Vector Laboratories, Burlingame, CA, USA). For double fluorescent staining, fixed brain tissues were soaked in anti-MPO (1:200) antibody solutions overnight at 4 °C. After washing three times with PBS, sections were incubated with rhodamine-conjugated anti-rabbit IgG (1:200, Jackson ImmunoRes Lab, West Grove, PA, USA) in PBS for 1 h at 20–22 °C. Brain sections were counterstained with 4′,6-diamidino-2-phenylindole (DAPI) to visualize nuclei, and observed under a fluorescence microscope (Axioplan 2, Zeiss, Oberkochen, Germany).

### 2.9. BV2 Cell Culture

BV2 cells, murine microglial cells, were obtained from ATCC (Manassas, VA, USA); grown in DMEM supplemented with 10% FBS, 100 U/mL penicillin and 100 μg/mL streptomycin; and maintained at 37 °C in a 5% CO_2_ incubator.

### 2.10. RNA Preparation and qRT-PCR

Total RNA was extracted from BV2 cells and brain slices of cerebral cortex using a total RNA isolation (TRI) reagent (MRC, Cincinnati, OH, USA), and then reverse transcribed according to the manufacturer’s protocol (Takara Bio, Tokyo, Japan). Then, quantitative PCR was performed using a real time PCR detection system (BioRad, Hercules, CA, USA) with the SYBR Green PCR Master Mix (Toyobo, Osaka, Japan) and the following primers (forward and reverse, respectively): GCLC, 5′-CCT TCT GGC ACA GCA CGT TG-3′ and 5′-TAA GAC GGC ATC TCG CTC CT-3′; HO-1, 5′-AAG CCG AGA ATG CTG AGT TCA-3′ and 5′-GCC GTG TAG ATA TGG TAC AAG GA-3′; NQO1, 5′-AGA GGC TCT GAA GAA GAG AGG-3′ and 5′-CAC CCT GAA GAG AGT ACA TGG-3′; Prx-1, 5′-CAC TGA CAA ACA TGG GGA AGT–3′ and 5′-TTT GCT CTT TTG GAC ATC AGG–3′; MPO, 5′-ACCTACCCCAGTACCGATCC-3′ and 5′-AACTCTCCAGCTGGCAAAAA-3′; GAPDH, 5′-CCT TCC GTG TTC CTA CCC C-3′ and 5′-CCC AAG ATG CCC TTC AGT-3′.

### 2.11. Western Blotting

BV2 cell lysates were prepared in a lysis buffer containing 20 mM Tris-HCl (pH 8.0), 150 mM NaCl, 1 mM EDTA, 1% Triton X-100, 20 μg/mL chymostatin, 10 μM leupeptin, and 2 mM phenylmethylsulfonyl fluoride (PMSF) as described previously [36]. Brain slices from cerebral cortex were lysed in radioimmunoprecipitation assay buffer (50 mM Tris-HCl, pH 7.4, 1% NP-40, 0.25% sodium deoxycholate, 150 mM NaCl) containing 20 μg/mL chymostatin, 10 uM leupeptin, and 2 mM PMSF. Total protein (20–30 μg) was separated by SDS-PAGE. The resolved proteins were transferred onto polyvinylidene fluoride membranes (Millipore, Bedford, MA, USA), and the blots were probed with specific antibodies and developed using a chemiluminescence kit (Amersham, Arlington Heights, IL, USA). Integrated densitometry was used to determine the intensity of scanned films using Image J software (NIH, Bethesda, MD, USA).

### 2.12. Statistical Analysis

Two-sample comparisons were performed using the Student–Newman–Keuls test, multiple comparisons were performed using one-way analysis of variance (ANOVA), and two-tailed Student’s *t*-test was performed with Prism 5.0 software (GraphPad, San Diego, CA, USA). Results are presented as mean ± standard error of the mean (SEM) or mean ± standard deviation (SD), and *p* < 0.05 was considered statistically significant.

## 3. Results

### 3.1. Intranasally Delivered Tau-Cl Suppressed Infarct Formation in the Rat MCAO Model

**Figure 1:**
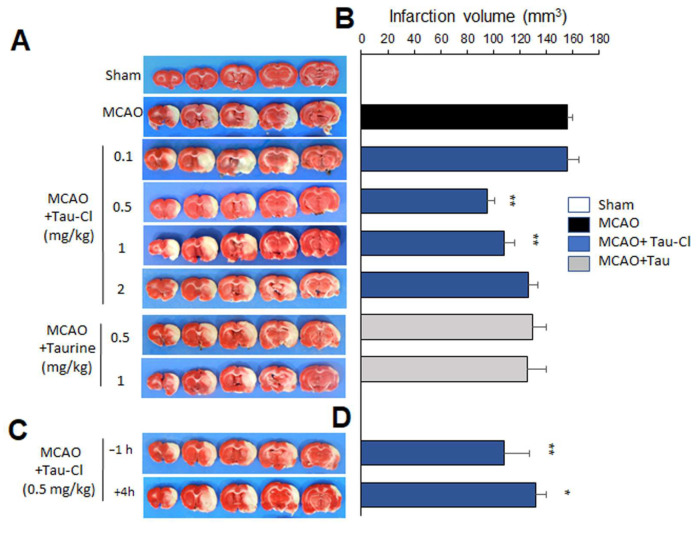
Tau-Cl suppressed infarct formation in the postischemic brain. (**A**,**B**) Tau-Cl (0.1, 0.5, 1 or 2 mg/kg) or taurine (0.5 or 1 mg/kg) was administered intranasally at 1 h post-middle cerebral artery occlusion (MCAO) and 2,3,5-triphenyl tetrazolium chloride (TTC) staining was carried out at 2 days post-MCAO. (**C**,**D**) Tau-Cl (0.5 mg/kg) was administered intranasally at 1 h prior to or 4 h post-MCAO, and TTC staining was carried out at 2 days post-MCAO. Representative images of infarctions are shown (**A**,**C**), and mean infarction volumes are presented as mean ± SEM (n = 4–10) (**B**,**D**). Figure 1C shares the same control group as Figure 1A. Sham—sham-operated rats (n = 4); MCAO—phosphate-buffered saline (PBS)-treated MCAO control (n = 10); MCAO + Tau-Cl—Tau-Cl-treated MCAO rats (n = 33); MCAO + taurine—taurine-treated MCAO rats (n = 8). * *p* < 0.05, ** *p* < 0.01 vs. PBS-treated MCAO control by one-way ANOVA with Student–Newman–Keuls test.

Neuroprotective effects of taurine have been reported in previous studies, wherein taurine was administered intraperitoneally [31] or intravenously [7,8,28] at 30 min prior to [31] or 1 to 24 h post-MCAO [7,8,28]. To compare the neuroprotective potency of Tau-Cl with that of taurine in the postischemic brain, we administered Tau-Cl (0.1, 0.5, 1, or 2 mg/kg) to a MCAO rat model intranasally at 1 h post-MCAO and examined the infarct volume at 48 h after surgery. Although Tau-Cl had no effect at 0.1 mg/kg, the infarct volume was significantly reduced by the administration of 0.5 mg/kg Tau-Cl and was 61.2 ± 5.4% (n = 8, *p* < 0.01) of that of PBS-treated MCAO controls (Figure 1A,B). A similar level of reduction (69.5 ± 6.1%, n = 8, *p* < 0.01) was detected with 1 mg/kg of Tau-Cl (Figure 1A,B), indicating a robust neuroprotective effect of Tau-Cl in the postischemic brain. However, no significant reductions in the mean infarct volume were detected with 2 mg/kg of Tau-Cl (Figure 1A,B), indicating that its neuroprotective potency was not dose-dependent. Interestingly, no significant reductions in the mean infarct volume were detected in the MCAO + Taurine groups. The administration of 0.5 or 1 mg/mL taurine (1 h post-MCAO, intranasal) failed to reduce mean infarct volumes 81.3 ± 8.1% (n = 5) and 78.2 ± 8.8% (n = 4), respectively, compared with those of PBS-treated MCAO controls (Figure 1A,B). When Tau-Cl (0.5 mg/kg) was administered intranasally 1 h prior to MCAO, the mean infarct volume was significantly reduced (69.7 ± 9.3%, n = 4, *p* < 0.05) at 2 days post-MCAO, and the administration of Tau-Cl at 4 h post-MCAO also reduced infarct volume (84.5 ± 5.4%, n = 7, *p* < 0.05) (Figure 1C,D). These results indicate that Tau-Cl exerts neuroprotective effects in the postischemic brain with a wide therapeutic window.

### 3.2. Tau-Cl Improved Neurological Deficits and Motor Impairment in the Rat MCAO Model

**Figure 2:**
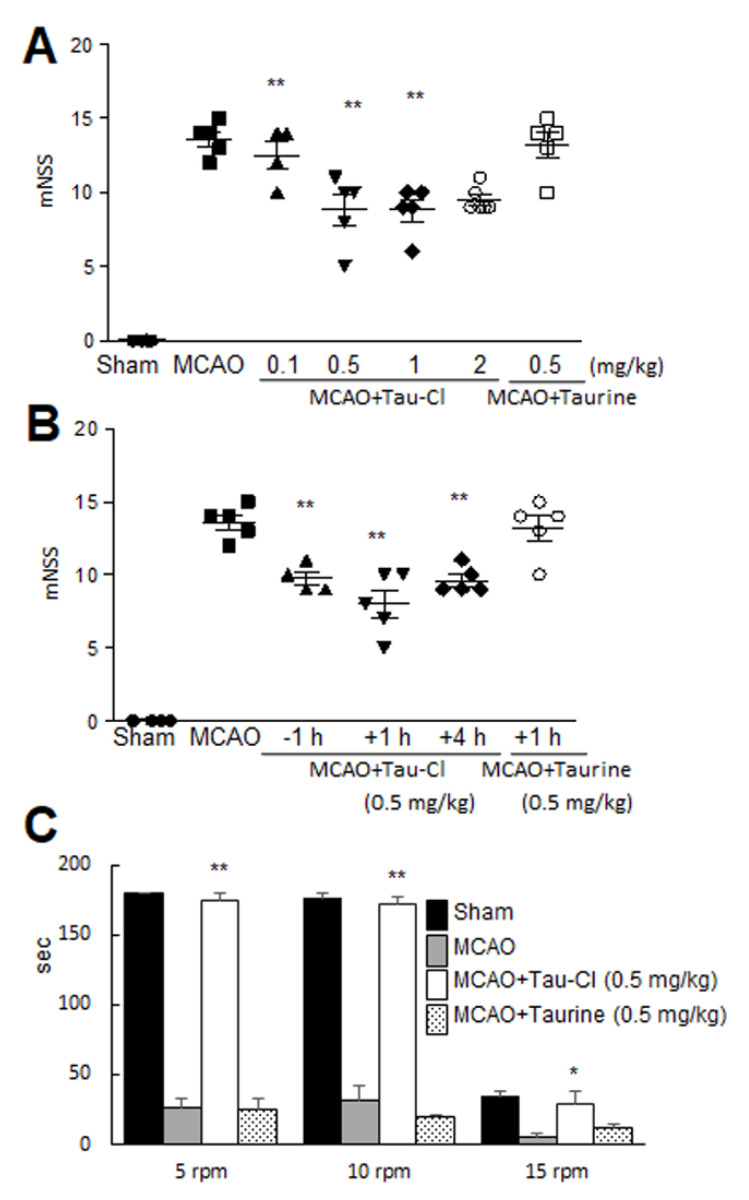
Tau-Cl recovered neurological and motor deficits. (**A**,**B**) Tau-Cl (0.1, 0.5, 1 or 2 mg/kg) or taurine (0.5 mg/kg) was administered intranasally 1 h post-MCAO (**A**), or Tau-Cl (0.5 mg/kg) was administered intranasally at 1 h prior to or 1 or 4 h post-MCAO (**B**). Neurological deficits were evaluated using modified neurological severity scores at 2 days post-MCAO. (**C**) Tau-Cl (0.5 mg/kg) or taurine (0.5 mg/kg) was administered intranasally 1 h post-MCAO, and the rotarod test was performed 2 days post-MCAO at 5, 10, and 15 rpm at with a 1 h inter-trial rest period. Data are presented as mean ± SEM (n = 3–6). Sham, sham-operated rats (n = 4); MCAO, PBS-treated MCAO controls (n = 10); MCAO + Tau-Cl, Tau-Cl-treated MCAO rats (n = 10); MCAO + Taurine, taurine-treated MCAO rats (n = 8). * *p* < 0.05, ** *p* < 0.01 vs. PBS-treated MCAO controls by one-way ANOVA with Student–Newman–Keuls test.

To determine whether Tau-Cl can improve neurological deficits and motor impairment, 0.5 mg/kg of Tau-Cl was administered 1 h post-MCAO, and mNSSs were measured 2 days after MCAO. The mean mNSS at 2 days post-MCAO was 13.6 ± 0.5 (n = 5) for PBS-treated MCAO controls (Figure 2A). The mean mNSSs were significantly lower in groups receiving 0.5 or 1 mg/kg of Tau-Cl (8.8 ± 1.1, n = 5, *p* < 0.01 and 8.8 ± 0.7, n = 5, *p* < 0.01, respectively), compared to those of PBS-treated MCAO controls (Figure 2A), indicating significant improvement in neurological deficits. In contrast, the mean mNSS for the MCAO + Taurine group was not significantly different from that of the MCAO-PBS control group (Figure 2A). As expected, the mean mNSS of the two MCAO + Tau-Cl groups that were administered Tau-Cl 1 h prior to or 4 h post-MCAO were also significantly lower than the mean mNSS of the MCAO + PBS control group (Figure 2B). In addition, when motor activities were assessed at 2 days post-MCAO using the rotarod test at 5 rpm, the mean time spent on the rotarod by the MCAO + Tau-Cl (0.5 mg/kg, 1 h post-MCAO) group was almost the same as that by the sham control group (Figure 2C). At 10 rpm, the mean time spent on the rotarod by the MCAO + Tau-Cl group was significantly longer than that by PBS-treated MCAO controls and the taurine-treated MCAO group (Figure 2C). Interestingly, at 15 rpm, the mean time spent on the rotarod in MCAO + Tau-Cl group was markedly shorter than those at 5 or 10 rpm, but it was still significantly higher than that by PBS-treated MCAO controls (Figure 2C). These results suggest that the neuroprotective effect of Tau-Cl was accompanied by the amelioration of motor impairment and neurological deficits. The physiological parameters pH, PaO_2_, PaCO_2_, and blood glucose were similar in the MCAO + Tau-Cl, MCAO + Taurine, and PBS-treated MCAO groups (Table 1).

### 3.3. Neutrophils Were Infiltrated into the Brain Parenchyma after Ischemic Insult

**Figure 3:**
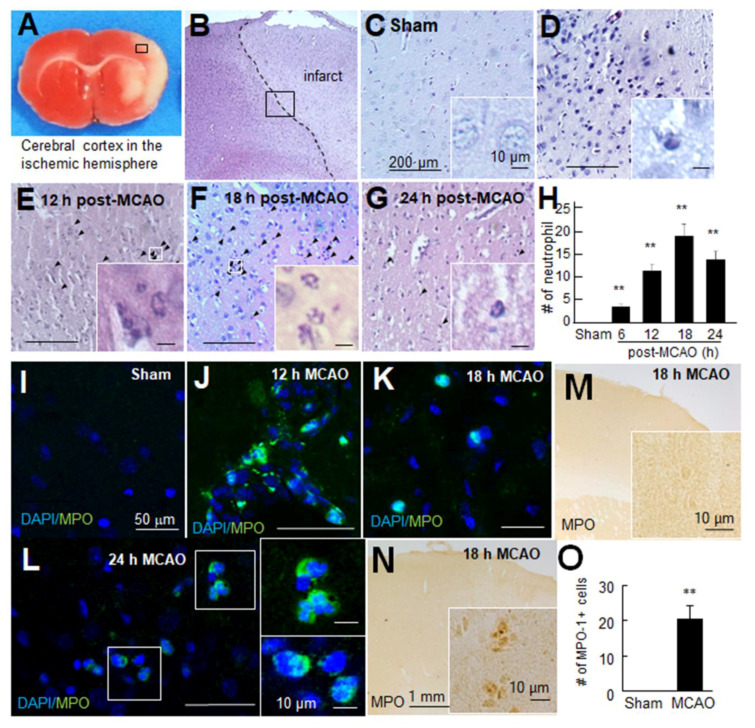
Neutrophils were infiltrated in the post-MCAO brains. (**A**–**H**) Coronal brain sections were prepared at 6, 12, 18, or 24 h post-MCAO and stained with Hematoxylin and Eosin. Numbers of neutrophils were counted in the cerebral cortex indicated by the black box (0.5 × 0.5 mm^2^). Representative pictures are shown (**B**–**G**), and results are presented as mean ± SEM (n = 12 from 3 animals) (**H**). (**I**–**L**) Coronal brain sections were prepared from the sham-operated control group and MCAO group at 12, 18, or 24 h post-MCAO and stained with anti-myeloperoxidase (MPO) antibody and 4′,6-diamidino-2-phenylindole (DAPI). Representative pictures of the cortex are shown. (**M**–**O**) Coronal brain sections were prepared from the sham control group and MCAO group at 18 h post-MCAO and stained with anti-MPO antibody, and numbers of MPO-positive cells were counted in the indicated black box (0.5 × 0.5 mm^2^). Scale bars in (**C**–**G**) and (**I**–**L**) represent 200 and 50 μm, respectively, and those in insets represent 10 μm. Sham, sham-operated rats; MCAO, PBS-treated MCAO controls. ** *p* < 0.01 vs. sham control by one-way ANOVA with Student–Newman–Keuls test.

Neutrophils are the first blood-derived cells to accumulate around damaged areas in the ischemic brain [37], and the conversion of taurine to Tau-Cl is known to be mediated by MPO, which is mainly provided by neutrophils [17]. A robust neuroprotective effect of Tau-Cl, which is greater than that of taurine, prompted us to examine the temporal profile of neutrophil infiltration in the postischemic brain. Neutrophils can be easily identified by their multi-lobed nuclei after H&E staining. In sham controls, neutrophils were rarely detected throughout the brain, including the cerebral cortex indicated by the black box in Figure 3A–C). However, in PBS-treated MCAO controls, neutrophils were detected at 6 h post-MCAO, and their numbers increased until 18 h post-MCAO and then subsided (Figure 3D–H). Double fluorescent staining of brain samples obtained at 12, 18, or 24 h post-MCAO with an anti-MPO antibody and DAPI showed that neutrophils were present in the brain parenchyma and intravascular and perivascular regions (Figure 3I–L). In addition, immunohistochemistry showed that the number of MPO-positive cells increased at 18 h post-MCAO, further supporting the infiltration of neutrophils into the ischemic cerebral cortex (Figure 3M–O).

### 3.4. Tau-Cl Increased the Expression of Antioxidant Enzymes in BV2 Microglia

**Figure 4:**
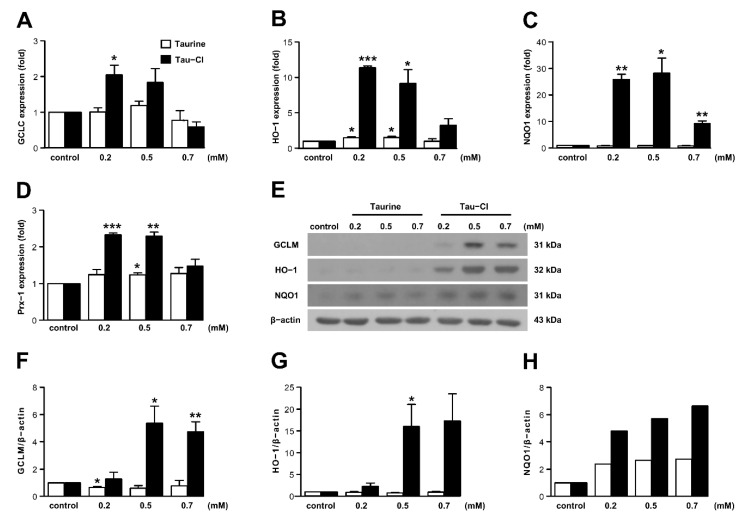
Tau-Cl increased the expression of antioxidant enzymes in BV2 cells. (**A**–**D**) BV2 cells were treated with various concentrations of taurine and Tau-Cl. The expressions levels of glutamate–cysteine ligase catalytic (GCLC) subunit (**A**), heme oxygenase-1 (HO-1) (**B**), NADPH:quinone oxidoreductase-1 (NQO1) (**C**), and peroxiredoxin-1 (Prx-1) (**D**) mRNA were determined by qRT-PCR (n = 4). (**E**–**H**) The protein levels of glutamate–cysteine ligase modifier (GCLM) subunit, HO-1, and NQO1 were determined by immunoblotting, and blots quantified by using the Image J software are presented as arbitrary density units (**F**–**H**) (n = 3). A representative arbitrary density of NQO1 from one of three independent experiments is shown in (**H**). Results are presented as mean ± SEM, * *p* < 0.05, ** *p* < 0.01, and *** *p* < 0.01 vs. control by a two-tailed Student’s *t*-test.

Antioxidant enzymes eliminate ROS in the brains of patients with neuropathological diseases [38,39], and Tau-Cl can increase the levels of antioxidant enzymes [3,40]. In the present study, BV2 cells were employed to determine if the effects of Tau-Cl, shown in the MCAO model, occur through antioxidant activity. We determined the cellular levels of antioxidant enzymes GCLC, HO-1, NQO1, and Prx-1 in BV2 cells, and evaluated the effect of taurine and Tau-Cl on the expression of these antioxidant enzymes. The maximum HO-1 mRNA and protein expressions were reached at 6–12 and 12 h, respectively (data not shown). We therefore treated BV2 cells for 6 h to measure mRNA expression, and for 12 h to measure protein expression. Tau-Cl increased the mRNA expression of antioxidant enzymes in BV2 cells compared to untreated control cells, and the highest expression was observed at concentrations between 0.2–0.5 mM (Figure 4A–D). Tau-Cl treatment resulted in significantly higher expression of HO-1 (11.4- and 9.2-fold) and NQO1 (25.8- and 28.2-fold) at 0.2 and 0.5 mM, respectively, compared to the control. Tau-Cl also increased the expression of GCLC (2.1- and 1.8-fold) and Prx-1 (2.3- and 2.3-fold) at 0.2 and 0.5 mM, respectively, compared to the control. Taurine slightly but significantly increased HO-1 (1.5-fold at 0.2 and 0.5 mM) and Prx-1 (1.2-fold at 0.5 mM) expression, which may mean the effect of the conversion of taurine to Tau-Cl. However, taurine had no significant effect on GCLC and NQO1 expression.

Consistent with the mRNA expression, Tau-Cl increased the expression of antioxidant proteins GCLM, HO-1, and NQO1 compared to the control; however, taurine had no significant effect on the expression of these proteins (Figure 4E–H). In addition, although the mRNA levels were highest 0.2–0.5 mM Tau-Cl, protein expression levels remained elevated up to 0.7 mM Tau-Cl. Protein expression of Prx-1 was not detected, which supports the previous finding that Prx-1 is expressed moderately in microglia but apparently in oligodendrocytes [41], whereas Prx-5 is highly expressed in macrophages [42].

### 3.5. Tau-Cl Increased Antioxidant Enzyme Expression in a Rat MCAO Model

**Figure 5:**
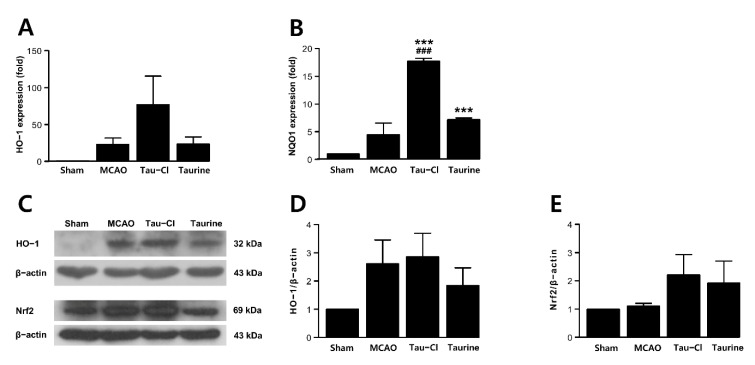
Tau-Cl increased the expression of antioxidant enzymes in the post-MCAO brains. Brain samples for qRT-PCR and immunoblot analysis were prepared from cerebral cortex indicated in Figure 3A at 24 h post-MCAO. (**A**,**B**) The expression levels of mRNA of HO-1 (**A**) and NQO1 (**B**) were determined by qRT-PCR (n = 3). (**C**–**E**) The protein levels of HO-1 and Nrf2 were determined by immunoblotting (**C**), and blots quantified by using the Image J software are presented as arbitrary density units (**D**,**E**) (n = 3). Results are presented as mean ± SEM, *** *p* < 0.001 vs. sham control, and ^###^ *p* < 0.001 vs. PBS-treated MCAO controls by one-way ANOVA with Student–Newman–Keuls test.

Ischemic injury causes oxidative stress, which induces the expression of antioxidant genes as well as proinflammatory genes. The mRNA expression levels of HO-1 and NQO1 in the 24 h post-MCAO brain were increased (Figure 5A,B). Importantly, they increased further in the MCAO + Tau-Cl group, and the enhancement of NQO1 expression was also observed in the MCAO + Taurine group (Figure 5A,B), indicating that Tau-Cl exerts anti-oxidative effects in the postischemic brain. Similar results were obtained by immunoblot analysis of HO-1 and Nrf2, but the increase by Tau-Cl at the protein level was not as prominent as found at the mRNA level (Figure 5C–E). Together, these results indicate that the anti-oxidative effects of Tau-Cl were responsible, at least in part, for the observed robust neuroprotective effect in MCAO animals. Along with the presence of neutrophils, these effects might contribute to the previously reported neuroprotective effects of taurine.

## 4. Discussion

In the present study, we demonstrated a robust neuroprotective effect of Tau-Cl using an experimental stroke animal model and proposed that upregulation of anti-oxidative genes could be responsible for these effects. Although the beneficial effects of Tau-Cl have been reported in various pathological conditions, such as arthritis [43,44], testicular damage [45], and sepsis [46], to the best of our knowledge, this is the first report demonstrating a direct neuroprotective effect of Tau-Cl in cerebral ischemia. We showed that Tau-Cl at the doses of 0.5 or 1 mg/kg significantly suppressed infarct volume when they were delivered intranasally at 1 h post-MCAO. However, it is noteworthy that the effect was insignificant when Tau-Cl was administered at a dose of 2 mg/kg. This might be due to the cytotoxic effect of Tau-Cl at a high concentration reported in a previous study [32]. Tau-Cl (0.5 mg/kg) has a relatively wide therapeutic window, from 1 h prior to to 4 h post-MCAO.

In the ischemic brain, extracellular taurine levels are significantly increased following the release of intracellular taurine, which might scavenge HOCl generated after ischemic insult to produce Tau-Cl. Taurine can exert protective effects in animal models of stroke by reducing brain infarct volume, ameliorating morphological injury, and mitigating neurological deficits [6,8,29,31]. Several underlying molecular mechanisms have been proposed, including prevention of neuronal cell apoptosis [6,7,29,30]; activation of neuronal receptors, such as γ-aminobutyric acid type A and glycine receptors [6]; suppression of inflammation by inhibiting neutrophil infiltration and pro-inflammatory cytokine production [8,31]; restoring brain injury-induced antioxidants and increasing antioxidant capacity [30,31]; and preventing brain hemorrhage [14]. Based on the present study, we can speculate that the above-mentioned anti-oxidative effects of taurine are attributable to Tau-Cl. Taurine showed no protective effect at a dose of 0.5 or 1 mg/kg, which is 50–100-fold lower than the doses used in previous reports [6,7,30]. Tau-Cl therefore has a much greater anti-oxidative capacity than taurine. Notably, we also observed effective suppression of infarct formation by taurine when 10, 20, 50, or 100 mg/kg was administered intranasally 1 h prior to MCAO (unpublished data). However, it cannot be said that high concentrations of taurine have only positive effects. Because different doses of taurine (0.03, 2.8, and 5.6 g/kg) had opposite effects on dopamine transporter expression and dopamine uptake [47], and despite neuroprotective effects, high dose of taurine inhibited the proliferation and differentiation of neural cells [48].

The rolling and adhesion of neutrophils infiltrating in the ischemic brain begins at 2 h post-ischemia, which is followed by their accumulations within vessels near injured brain regions 6–8 h later and peak infiltration into brain tissue at 18–24 h post-ischemia [49,50]. The accumulated active neutrophils release ROS, granular enzymes, and cytokines and form neutrophil extracellular traps at the site of infarction [51]. These increase inflammation and chemoattraction/activation of adjacent immune cells, causing further damage to the blood–brain barrier and inducing the activation of microglial cells. Neutrophils contain a large amount of primary granule MPO, which catalyzes the formation of HOCl. MPO has been implicated in many brain diseases, including stroke, Alzheimer’s disease, and multiple sclerosis [52,53]. MPO is widely distributed in ischemic tissues and correlates positively with infarct size [52]. The inhibition of MPO markedly decreases infarct size and neuronal damage [54,55]. Accordingly, serum MPO levels are elevated in human stroke patients [56,57], and certain MPO genotypes are associated with increased brain infarct size and poor functional outcome in human cerebral ischemia [58]. Neutrophils and macrophages/microglia are known to contribute to the secretion of MPO in the ischemic brain, and the infiltration of neutrophils and macrophages/microglia in the infarct region was detected in the present study [50]. We compared the basal levels of MPO expression in murine bone marrow neutrophils, macrophage cell line RAW 264.7 cells, and BV2 cells, and murine bone marrow neutrophils expressed more than 50,000 times of MPO at the mRNA level compared to RAW 264.7 cells and BV2 cells (unpublished data), which suggests that neutrophils are the main source of MPO in the ischemic brain. However, many reports state that MPO is present, to a lesser extent, in macrophages/microglia. In particular, activated macrophages/microglia contain higher levels of MPO than unstimulated cells [59,60].

Taurine reacts stoichiometrically with HOCl to produce Tau-Cl by the MPO system, resulting in the elimination of the most powerful oxidant HOCl. In the present study, we showed neutrophil infiltration and the presence of MPO in the ischemic hemisphere of the postischemic brain (Figure 3). The large amount of taurine present in the brain scavenges ROS and generates Tau-Cl. In addition to this ROS scavenging activity, Tau-Cl possesses its own anti-inflammatory and anti-oxidative properties. Tau-Cl inhibits the overproduction of inflammatory mediators, such as nitric oxide, tumor necrosis factor-α, prostaglandins, interleukins, macrophage inflammatory protein, monocyte chemoattractant protein, and matrix metalloproteinases. Tau-Cl plays an anti-oxidative role by inhibiting ROS production and increasing the levels of antioxidants, such as glutathione, HO-1, GPx, Prx, and catalase [3,40]. Although we do not know exactly how many neutrophils infiltrate and how much MPO and Tau-Cl are produced in the ischemic brain, the neuroprotective effect of taurine appears to be closely linked to Tau-Cl.

In the present study, exogenously applied Tau-Cl suppressed infarct formation and improved neurological deficits and motor impairment to a greater degree than that by taurine in a rat MCAO model. Tau-Cl increased the expression of antioxidant enzymes in a dose-dependent manner in BV2 cells, whereas taurine had a little effect. At 24 h post-MCAO, the expression of antioxidants was induced, and Tau-Cl treatment during the acute phase (1 h post-MCAO) further enhanced this induction, possibly mitigating ischemic brain damage. However, contradictory results have been reported regarding the expression and activation of antioxidant enzymes in MCAO models, with some reporting increasing [61,62] and some decreasing expressions [63,64]. These contradictions might be caused by the experimental conditions, such as reperfusion time and brain region assayed.

## 5. Conclusions

In summary, Tau-Cl reduced MCAO-induced infarct volume and improved neurological deficits and motor impairment in a rat MCAO model. Tau-Cl increased the expression of antioxidant enzymes in BV2 cells and further enhanced the induction of the same antioxidant enzymes in the postischemic brain. These results suggest that taurine protects against ischemic brain injury via Tau-Cl, which inhibits proinflammatory mediators and increases the levels of antioxidants. Thus, although there are no antioxidant-based drugs to date, stroke improvement by increased antioxidant enzymes provides clues to a new concept in stroke treatment.

## Figures and Tables

**Table 1:** Physiological parameters.

	Vehicle-Treated Group (n = 3)		Tau-Cl-Treated Group (n = 3)	
	Base	During Ischemia	Base	During Ischemia
Rectal Temperature (°C)	36.1 ± 0.3 *	36.5 ± 0.5	36.0 ± 0.6	36.1 ± 0.2
pH	7.5 ± 0.1	7.4 ± 0.1	7.5 ± 0.1	7.4 ± 0.1
PO₂ mmHg	82.3 ± 4.6	89.0 ± 12.2	91.0 ± 10.6	74.7 ± 0.6
PCO₂ mmHg	34.3 ± 7.4	43.8 ± 3.0	36.9 ± 6.1	36.2 ± 4.7
Glucose, mg/dL	111.3 ± 12.1	115.7 ± 7.1	106.7 ± 3.1	107.3 ± 3.1

* Values are means ± SD (n = 3). One-way ANOVA revealed no significant intergroup difference for any variance.

## Data Availability

The data presented in this study are available on request from the corresponding author.
