# Correction: Seol et al. Taurine Protects Against Postischemic Brain Injury via the Antioxidant Activity of Taurine Chloramine. *Antioxidants* 2021, *10*, 372

**DOI:** 10.3390/antiox15040418

**Published:** 2026-03-27

**Authors:** Song-I Seol, Hyun Jae Kim, Eun Bi Choi, In Soon Kang, Hye-Kyung Lee, Ja-Kyeong Lee, Chaekyun Kim

**Affiliations:** 1 Department of Anatomy, Inha University School of Medicine, Incheon 22212, Republic of Korea; ssie8878@naver.com (S.-I.S.); marrina@hanmail.net (H.-K.L.); 2 BK21, Program in Biomedical Science & Engineering, Inha University, Incheon 22212, Republic of Korea; zzang4128z@naver.com (H.J.K.); chldmsql777@naver.com (E.B.C.); 3 Laboratory of Leukocyte Signaling Research, Department of Pharmacology, Inha University School of Medicine, Incheon 22212, Republic of Korea; round001@hanmail.net; 4 Convergent Research Center for Metabolism and Immunoregulation, Inha University, Incheon 22212, Republic of Korea

In the original publication [1], there was a mistake in Figure 1C. The image representing the −1 h (pre-treatment) group was mistakenly duplicated from the +4 h (post-treatment) group. The image at the +4 h point is correct; however, the image at the −1 h point was incorrectly placed during figure assembly.

We have prepared a corrected Figure 1 and its legend (below). This figure incorporates the correct image for the −1 h group, selected from our original four images available for this experimental group. Additionally, to enhance clarity and avoid unnecessary repetition, we have simplified Figure 1C,D by removing redundant images, as all TTC images in these panels originated from the same set of experiments.

We confirmed that no changes had been made to the Results section in the manuscript. The data displayed in the corrected figure panels were already used for the statistical analysis, and we have confirmed that all statistics are correct. The authors confirm that this correction does not affect the scientific conclusions of the article. This correction was approved by the Academic Editor. The original publication has also been updated.

## Figures and Tables

**Figure 1:**
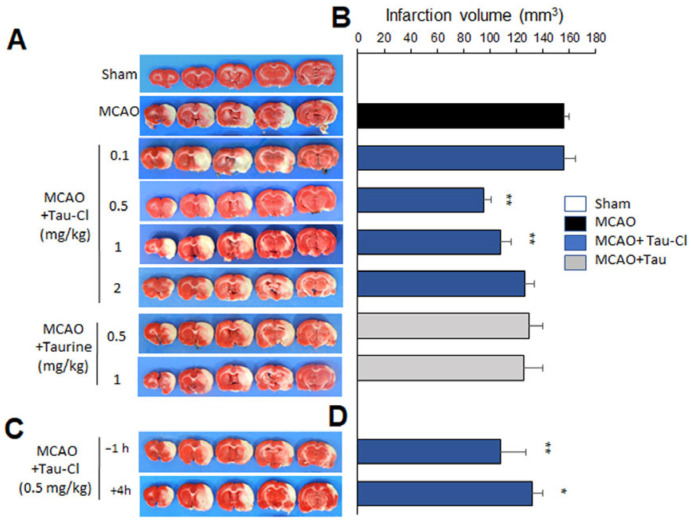
Tau-Cl suppressed infarct formation in the postischemic brain. (**A**,**B**) Tau-Cl (0.1, 0.5, 1 or 2 mg/kg) or taurine (0.5 or 1 mg/kg) was administered intranasally at 1 h post-middle cerebral artery occlusion (MCAO) and 2,3,5-triphenyl tetrazolium chloride (TTC) staining was carried out at 2 days post-MCAO. (**C**,**D**) Tau-Cl (0.5 mg/kg) was administered intranasally at 1 h prior to or 4 h post-MCAO, and TTC staining was carried out at 2 days post-MCAO. Representative images of infarctions are shown (**A**,**C**), and mean infarction volumes are presented as mean ± SEM (n = 4–10) (**B**,**D**). Figure 1C shares the same control group as Figure 1A. Sham—sham-operated rats (n = 4); MCAO—phosphate-buffered saline (PBS)-treated MCAO control (n = 10); MCAO + Tau-Cl—Tau-Cl-treated MCAO rats (n = 33); MCAO + taurine—taurine-treated MCAO rats (n = 8). * *p* < 0.05, ** *p* < 0.01 vs. PBS-treated MCAO control by one-way ANOVA with Student–Newman–Keuls test.
